# Combination of Enrichment Using Gene Ontology and Transcriptomic Analysis Revealed Contribution of Interferon Signaling to Severity of COVID-19

**DOI:** 10.1155/2022/3515001

**Published:** 2022-04-11

**Authors:** Hilmi Farhan Ramadhani, Annisa Annisa, Aryo Tedjo, Dimas R. Noor, Wisnu Ananta Kusuma

**Affiliations:** ^1^Department of Computer Science, Faculty of Mathematics and Natural Sciences, IPB University, Bogor 16680, Indonesia; ^2^Department of Medical Chemistry, Faculty of Medicine, Universitas Indonesia, Jakarta, Indonesia; ^3^Human Cancer Research Center, Indonesian Medical Education and Research Institute, Faculty of Medicine, Universitas Indonesia, Jakarta, Indonesia; ^4^Tropical Biopharmaca Research Center, IPB University, Bogor 16128, Indonesia

## Abstract

**Introduction:**

The severity of coronavirus disease 2019 (COVID-19) was known to be affected by hyperinflammation. Identification of important proteins associated with hyperinflammation is critical. These proteins can be a potential target either as biomarkers or targets in drug discovery. Therefore, we combined enrichment analysis of these proteins to identify biological knowledge related to hyperinflammation. Moreover, we conducted transcriptomic data analysis to reveal genes contributing to disease severity.

**Methods:**

We performed large-scale gene function analyses using gene ontology to identify significantly enriched biological processes, molecular functions, and cellular components associated with our proteins. One of the appropriate methods to functionally group large-scale protein-protein interaction (PPI) data into small-scale clusters is fuzzy K-partite clustering. We collected the transcriptomics data from GEO Database (GSE 164805 and GPL26963 platform). Moreover, we created a data set and analyzed gene expression using Orange Data-mining version 3.30. PPI analysis was performed using the STRING database with a confidence score >0.9.

**Results:**

This study indicated that four proteins were associated with 25 molecular functions, three were associated with 22 cellular components, and one was associated with ten biological processes. All GOs of molecular function, cellular components, and 9 of 14 biological processes were associated with important cytokines related to the COVID-19 cytokine storm present in the resulting cluster. The expression analysis showed the interferon-related genes IFNAR1, IFI6, IFIT1, and IFIT3 were significant genes, whereas PPIs showed their interactions were closely related.

**Conclusion:**

A combination of enrichment using GOs and transcriptomic analysis showed that hyperinflammation and severity of COVID-19 may be caused by interferon signaling.

## 1. Introduction

A coronavirus is a group of viruses from the subfamily Orthocoronavirinae in the Coronaviridae family and the order Nidovirales. This group of viruses can cause disease in birds and mammals, including humans [1]. In humans, coronaviruses cause respiratory infections that are generally light, such as colds, to some severe infections of the respiratory, digestive, and systemic systems [2]. Several forms of disease caused by this virus are Severe Acute Respiratory Syndrome (SARS) in 2002 and Middle East Respiratory Syndrome (MERS) in 2012. At the end of 2019, the Severe Acute Respiratory Syndrome Coronavirus-2 (SARS-CoV-2) caused a new disease named Coronavirus Disease 2019 (COVID-19) by the World Health Organization (WHO) on February 11, 2020. COVID-19 can cause significant health problems such as fever, dry cough, difficulty breathing, pneumonia, multi-organ failure, and even death [3]. In addition, the SARS-CoV-2 virus becomes a parasite on host cells and will cause an excessive inflammatory reaction or hyperinflammation [4].

The meaning of hyperinflammation is an excessive immune response that can cause high levels of inflammation. The excessive inflammation caused by this virus occurs due to a storm of cytokines that can damage the human lungs [5], even forcing immune cells to destroy healthy cells. Therefore, COVID-19 patients must receive intensive care [6]. In inflammation, protein can be a biomarker of organ damage [4].

In recent decades, the development of high-throughput experimental research and the availability of a wide variety of databases have led to the development of methods to explain systems biology [7]. Systems biology is an integrated field that links molecular components within the same biological and across different scales (e.g. cells, tissues, and organ systems) to physiological function and phenotype of organisms through quantitative reasoning, computational modelling, and high-performance experimental technology [7]. Systems biology methodology can be applied either as a bottom-up approach that gathers smaller functional units to create a system or as a top-down approach that starts from the overall view of the system and then tries to study smaller subsystems [7]. Systems biology uses both experimental and computational frameworks to answer biological questions. The computational task includes knowledge extraction using bioinformatics, statistical methods, and network analysis [7]. Network nodes are cellular components in systems biology, and edges are the reactions or interactions between these nodes. Considering cellular systems as networks is a valuable and practical way to understand the functional organization of cells by analyzing network topology [8, 9].

In a previous study, we implemented a bottom-up strategy using network analysis to investigate the important proteins from protein-protein interaction (PPI). We employed a clustering technique and topological measures, such as degree centrality, betweenness centrality, and closeness centrality [8], to identify several proteins that exacerbate COVID-19 from the effects of hyperinflammation [10].

As in the bottom-up approach, in this study, we tried to know the role of those proteins in hyperinflammation by conducting enrichment analysis using gene ontology (GO). Enrichment analysis is a method for identifying the class of genes or proteins that are overexpressed in a large set of genes or proteins and may be associated with the phenotype of the disease. The screening of important proteins requires analysis using gene ontology (GO) due to the impact of hyperinflammation caused by SARS-CoV-2. GO is one of the data sources for functional genomic research [8], which consists of three distinct aspects describing the function of proteins. Ontology is a tool for seeking biological knowledge by associating data (genes or gene products) with biological processes, molecular functions, and cellular components [11]. We also could get the advantage of reducing the false positives value by using GO data [12]. The use of biological information could get better results in identifying important proteins [13] because it can improve the interpretation of results [8]. However, research on this topic is still limited. Most studies ignore the biological meaning of proteins in the context of PPI [14].

Finding the association of proteins and GO can be conducted using clustering. One of the clustering technique, the fuzzy k-partite clustering method, can cluster large-scale PPI data into functionally small-scale clusters. In a previous study, fuzzy k-partite clustering was developed and used to perform tripartite clustering on disease-gene-protein [15]. Thus, in this case, we could use fuzzy k-partite clustering to group protein/gene and three GO components. As known, each biological network was multifunctional so that proteins could fit into more than one cluster [15]. In this study, we employed the fuzzy k-partite clustering approach to perform enrichment analysis of protein and GO. We also continued this enrichment results by conducting transcriptomic analysis from the GEO database. We expected to reveal particular genes contributing to the disease severity.

## 2. Methods

### 2.1. Data

This study used protein data that affect hyperinflammation in COVID-19. All proteins in this study are proteins of humans. These proteins were obtained by using computational biology methods from important protein candidate data and PPI data [10]. The important protein candidates' data were obtained from OMIM (https://www.omim.org/), UniProt (https://www.uniprot.org/), and previous research about the COVID-19's protein [16–18]. From these various sources, there are 57 proteins obtained. Moreover, the PPI data were obtained from STRING (https://stringdb.org/). These 57 proteins interact with other proteins so the PPI data contain 357 proteins and 1686 interactions. This PPI data must go through the preprocessing stage, such as cleaning edge duplication and eliminating PPIs in small subgraphs that are not connected to the main protein interaction network. This step shows that the PPI data contain 222 proteins and 1239 interactions.

The screening of important proteins was carried out in two stages by calculating the overall centrality value using PCA and clustering with ClusterONE. The overall centrality value was calculated from the seven centrality measures (degree centrality, betweenness centrality, closeness centrality, subgraph centrality, eigenvector centrality, information centrality, and network centrality); the weights of each centrality were obtained from the eigenvalue resulting from PCA. Next, we reduced the graph to obtain the subgraph using the induced graph method. The amount of protein taken for subgraph formation is 10% of the total protein in the main graph. These proteins are linked to most of the proteins in the graph compared to the remaining 90%. Moreover, the top 10% proteins with the highest score of overall centrality contain 124 interactions formed by 22 proteins. By using ClusterONE, there were two clusters of this subgraph. The first cluster is the best because it had a higher density, quality, and average overall value and a lower *p*-value. There were 20 important proteins in the first cluster, namely STAT3, TYK2, IL6, STAT1, JAK1, STAT2, TBK1, RSAD2, OAS2, OAS1, MX2, MX1, ISG15, IRF7, IRF3, IFNAR1, IFIT3, IFIT1, IFI6, and DDX58.

**Figure 1:**
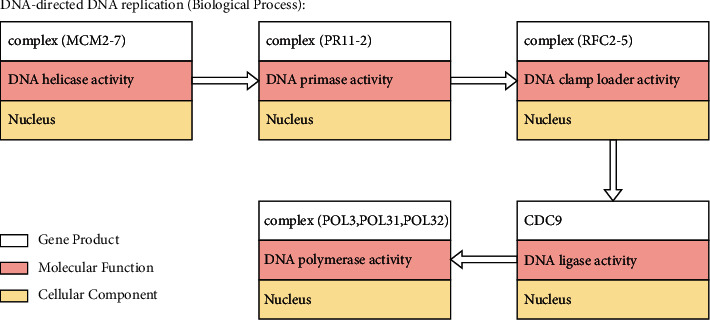
Illustration of three aspects in GO.

Moreover, we used GO data such as molecular function, cellular component, and biological process obtained from the UniProt database site. Three GOs represent GO terms describing a gene that encodes a gene product. These gene products carry out molecular-level activities (molecular functions) at specific locations relative to the cell (cellular components). These molecular-level processes contribute to a larger biological goal (biological processes) [11].  Figure 1 illustrates DNA replication in yeast modelled using three aspects of GOs. All GOs in this study are reviewed GOs in humans.

### 2.2. K-Partite Graph

In graph theory, a *k*-partite graph is a graph in which nodes can be divided into *k* independent sets. An independent set means that the nodes in a set are not connected to other nodes by an edge. In other words, no node is adjacent to another node in an independent set. In a *k*-partite graph, a node is adjacent to another node of a different set.

**Figure 2:**
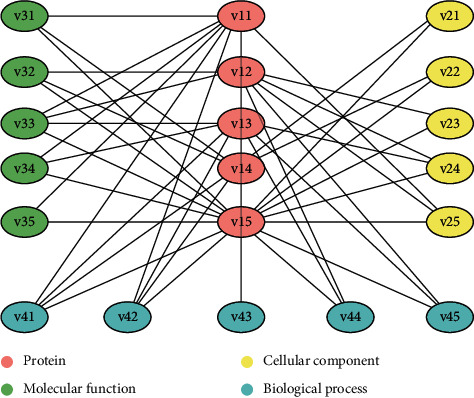
Illustration of three bipartite graph.

This study used four data, including protein and three GOs data (molecular function, cellular component, and biological process). Because the three GOs data are not related to each other, we could build three bipartite graphs, namely protein-molecular function, protein-cellular component, and protein-biological process. We used Cytoscape to visualize three bipartite graphs.  Figure 2 shows the illustration of bipartite graphs.

### 2.3. Fuzzy K-Partite Clustering

Fuzzy K-partite clustering applies the graph-based fuzzy clustering algorithm [15]. Fuzzy clustering is one of the methods to determine the optimal cluster in a vector space based on the Euclidean distance. It is a soft clustering method that can group an object into more than one cluster. In other words, fuzzy clustering can perform overlap clustering. The term fuzzy in the context of clustering is that each object has a degree of membership value to determine the cluster position of the object [19]. The purpose of fuzzy clustering is to minimize the objective function with the main parameter is the degree of membership [20]. So, the initial stage in this method is to determine the number of initial clusters. The determination of the maximum number of clusters for each GO data and protein can be seen in equations (1) and (2), respectively [15].

(1) $$\begin{array}{c} C_{\text{go}}=\frac{N_{\text{go}}}{10}, \end{array}$$

where *C*_go_ is the maximum number of clusters for each GO and *N*_go_ is the number of nodes in each GO data.

(2) $$\begin{array}{c} C_{p}=C_{\text{go}}\sqrt{\frac{N_{p}}{N_{\text{go}}}}, \end{array}$$

where *C*_*p*_ is the maximum number of protein clusters and *N*_*p*_ is the number of nodes in the protein data.

The fuzzy K-partite clustering algorithm inputs are matrix A, matrix B, and matrix C. Matrix A is the adjacency matrix between protein and GO. Matrix A was obtained from transforming three bipartite graphs into three adjacency matrices for each protein-molecular function graph, protein-cellular component graph, and protein-biological process graph. Element of matrix A has value one if an edge connects a protein node and a GO node and zero otherwise.

**Figure 3:**
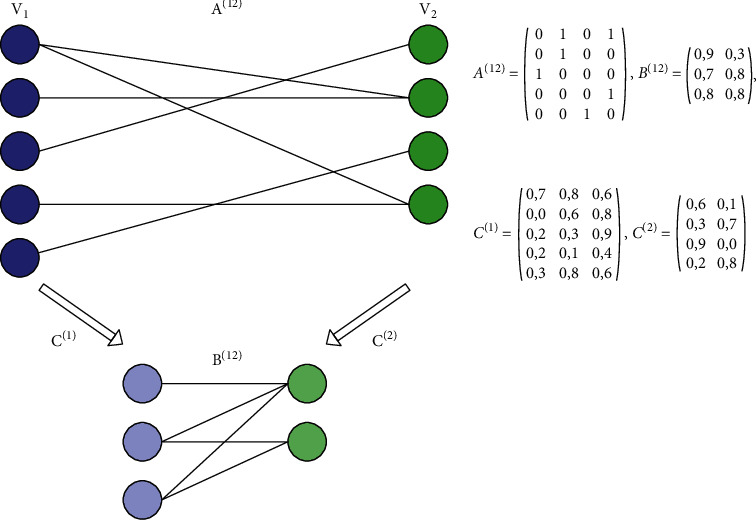
Matrix illustration on fuzzy K-partite clustering algorithm.

Matrix B is a matrix of interconnection value between protein clusters and GO clusters, while matrix C is a matrix of protein membership degree value in the protein cluster and GO in the GO cluster. In the fuzzy K-partite clustering algorithm, matrix B and matrix C are non-negative matrices whose initial value for each element is a random value. The dimensions of matrix B and matrix C depend on the number of proteins, the number of GO, the maximum number of protein clusters, and the maximum number of GO clusters.  Figure 3 illustrates matrix A, matrix B, and matrix C.

Figure 3 shows two sets, including the proteins and GO sets. The clustering process is carried out in three stages, as many as GO types. We conducted three clustering processes: clustering of important proteins with molecular function, cellular component, and biological process because there is no information about the relationship between each type of GO.

The output of this algorithm is the value of protein and GO membership degree in each cluster and the interconnection value between protein clusters and GO clusters. The interconnection value between clusters was high if the percentage of cluster members was low and vice versa. The fuzzy K-partite clustering algorithm will stop if the cost function value has converged. The cost function equation is calculated by equation (3) [15].

(3) $$\begin{array}{c} f\left(H,C\right)≔\sum_{i<j}{\left\|A^{\left(ij\right)}-C^{\left(i\right)}B^{\left(ij\right)}C^{\left(j\right)T}\right\|}_{F}^{2}, \end{array}$$

where ‖·‖_*F*_^2^ is the Frobenius norm of squares, i.e., the sum squares of the matrix elements. The cost function value shows how easily data are grouped into several clusters, the easier the data are grouped into a cluster, the lower the cost function value will be.

**Figure 4:**
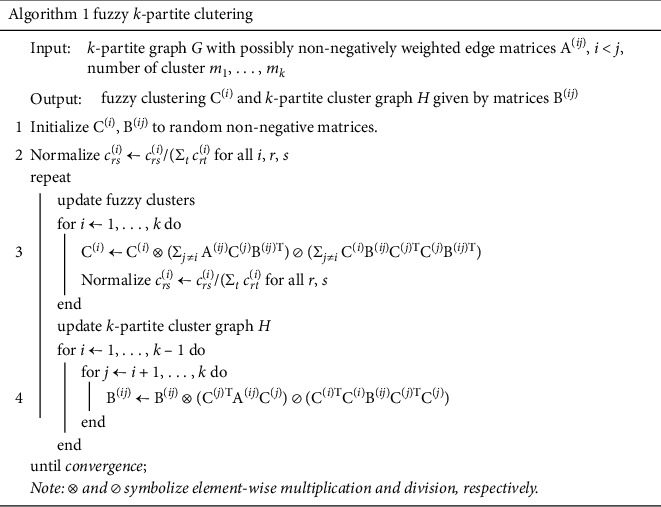
Fuzzy K-partite clustering algorithm.

U sing the fuzzy K-partite algorithm, the value of the cost function will find the lowest value because the algorithm's structure is similar to non-negative matrix factorization (NMF), with the difference that it can handle the factorization problem of three matrices. In addition, each iteration will not increase the cost function value. Another advantage is that fuzzy K-partite clustering produces a lower cost function value of 10% than usual and can predict the cluster structure better than the previous method since it is a soft clustering. The fuzzy K-partite clustering algorithm can be seen in Figure 4.

### 2.4. MicroArray Dataset Analysis

Microarray data were collected from data that referred to [21]. The data showed whole peripheral blood mononuclear cell (PBMC) genomic transcriptomes from severe (severe) and mild (mild) COVID-19 patients, as well as healthy controls (HC) retrieved from the GEO database (GSE 164805 and GPL26963 platform) [21]. Data set creation and gene expression analysis were performed using Orange Data-mining version 3.30.

### 2.5. PPI Analysis Using STRING

Protein-protein interaction (PPI) analysis of the altered expression of IRF7, IFNAR1, IFIT3, IFIT1, IFI6 in severe COVID-19 patients was performed using the STRING database. PPI analysis between the expression of these genes and the genes resulting from enrichment was carried out with a confidence score >0.9. The type of interaction, the confidence score, and the type of change in expression (upregulation or downregulation) were recorded and arranged in tabular form [22].

## 3. Result

### 3.1. Gene Ontology

We searched three GO data (cellular component, biological processes, and molecular function) from 20 proteins obtained from PPI analysis. There were 65 types of molecular function, 55 types of cellular component, and 274 types of biological processes associated with important proteins that have been obtained in the previous study.

**Figure 5:**
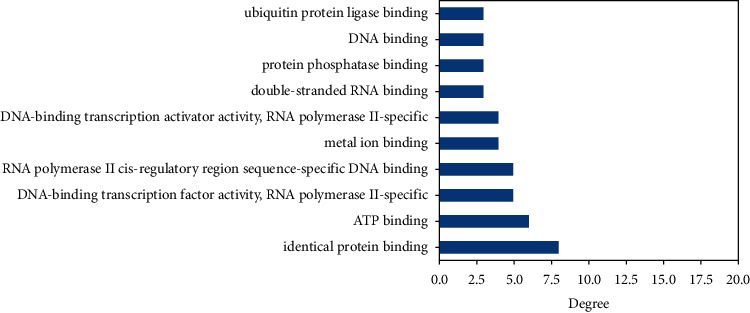
The highest degree of molecular function with important protein.

**Figure 6:**
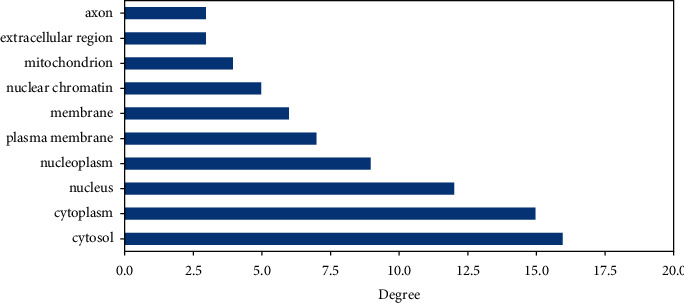
The highest degree of cellular component with important protein.

**Figure 7:**
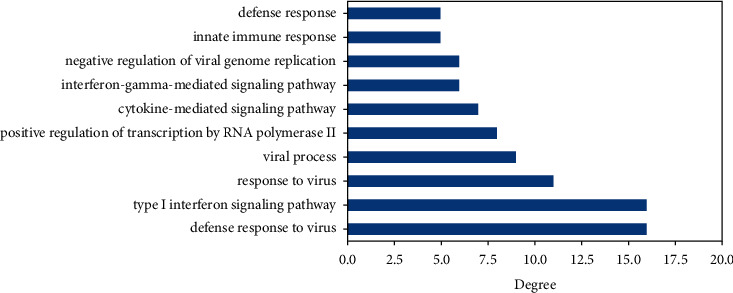
The highest degree of biological process with important protein.

Each GO data is associated with one important protein and has a relationship with more than one important protein. The GO data of molecular functions, cellular components, and biological processes that have the highest relationship or degree with the important proteins can be seen in Figures 5–7, respectively.

### 3.2. Bipartite Graph

**Figure 8:**
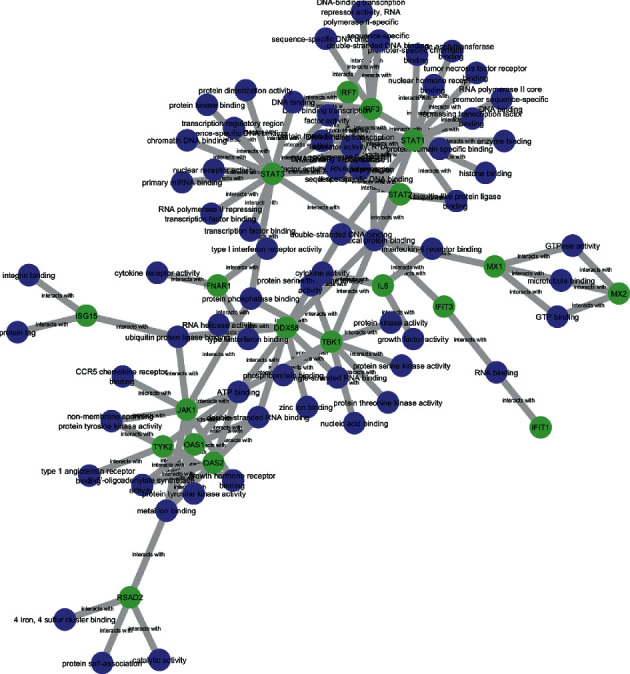
Bipartite graph of important protein-molecular function.

**Figure 9:**
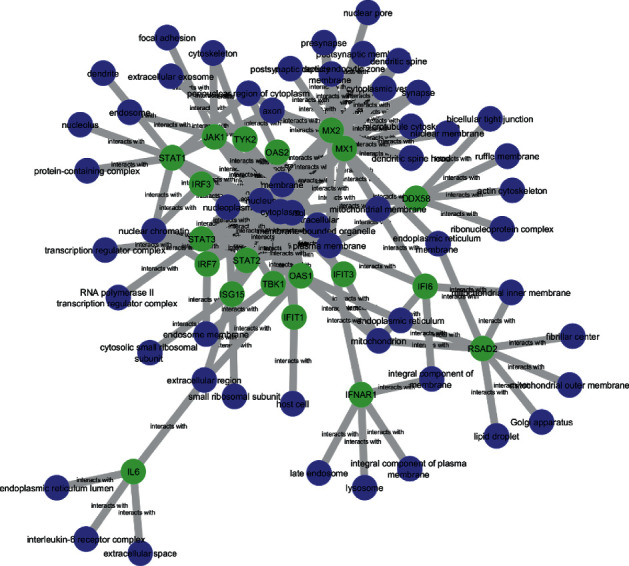
Bipartite graph of important protein-cellular component.

**Figure 10:**
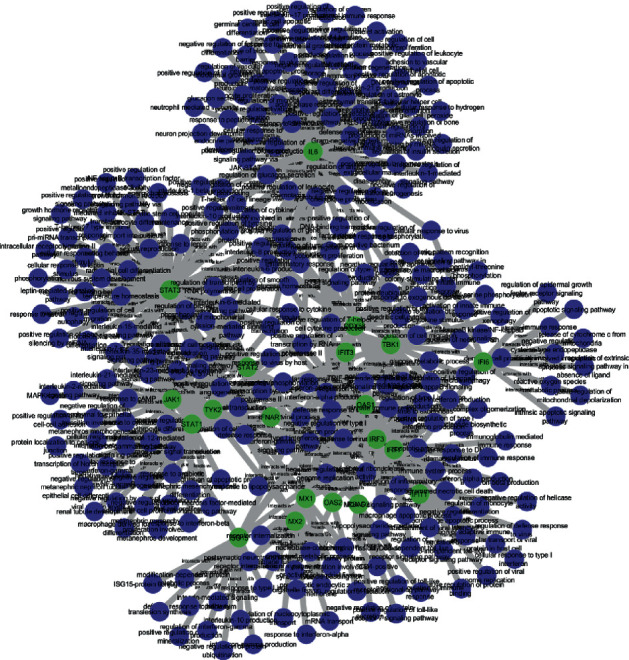
Bipartite graph of important protein-biological process.

Each GO is formed into a bipartite graph associated with important proteins using Cytoscape. There were 113 interactions between important proteins-molecular functions, 145 interactions between important proteins-cellular components, and 459 interactions between important proteins-biological processes. The illustrations of three bipartite graphs can be seen in Figures 8–10. The blue nodes are GO nodes, while the green nodes are important protein nodes.

### 3.3. Fuzzy K-Partite Clustering

After being formed into a bipartite graph, convert each graph into an adjacency matrix, or matrix A. Then calculate the maximum clusters formed for protein clusters in cellular components, molecular functions, and biological processes and each GO clusters of cellular components, molecular functions, and biological processes. With equations (1) and (2), the calculation results of the maximum clusters of each protein in cellular components, molecular functions, and biological processes and GO clusters of cellular components, molecular functions, and biological processes can be seen in Table 1.

After obtaining information on the maximum number of clusters in each protein and GO, matrix B and matrix C can be built with dimensions according to the maximum number of clusters in Table 1. This study did not search for the optimum cluster because the maximum number of clusters was relatively small. A protein or GO is assigned to a cluster if its membership degree exceeds the threshold of 0.2 [23].

### 3.4. Clustering Results Analysis

From the three results of bipartite graph clustering between important proteins with molecular function, cellular components, and biological processes, this study shows that four important proteins are associated with 25 molecular functions, three important proteins are associated with 22 cellular components, and one important protein associated with 101 biological processes. In addition, the resulting cost function values are 87.081 for molecular function, 109.985 for cellular components, and 311.371 for biological processes. These cost functions are smaller when compared to previous research, which had a cost function of 594.175 [24].

### 3.5. Significant Changes of Interferon-Associated Genes and Network Analysis

**Figure 11:**
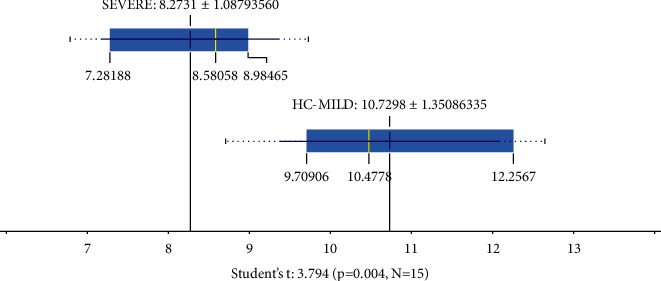
The mean IFNG expression in the SEVERE and MILD-HC groups was significantly different (*p* ≤ 0.01). It can be seen in the image that IFNG is downregulated that inhibits IFNG synthesis.

The interferon was found to be upregulated, including IFNAR1 (https://en.wikipedia.org/wiki/Interferon-alpha/beta_receptor), IFI6 (Interferon Alpha Inducible Protein 6), IFIT1 (Interferon Induced Protein With Tetratricopeptide Repeats 1), IFIT 3 (Interferon Induced Protein With Tetratricopeptide Repeats 3, IFNA6 (Interferon Alpha 6), and IFNB1 (Interferon beta precursor). On the other hand, the IRF7 (Interferon regulatory factor 7) and IFNG (Interferon Gamma) were found to be downregulated in severe COVID-19 patients. In Figure 11, we show the PPI interactions of these genes, IFI6, IFIT1, IFIT3, and IRF7, were strongly connected (confidence level = 0.9), while IFNAR was not connected. This shows that severity may be affected by changes of transcripts of interferon-related signaling.

From these data, we also observed the IFNG value in severe and mild-HC groups. The data are shown in Figure 11. The results showed that IFNG expression was downregulated significantly (*p* ≤ 0.01). Therefore, the severity of COVID-19 may be caused by the down expression of the IFNG. The results of gene expression analysis showed that there was a decrease (downregulation) of the expression of IFNG and IRF7, which was thought to affect the antiviral response by interferon in SEVERE patient's expressions.

**Figure 12:**
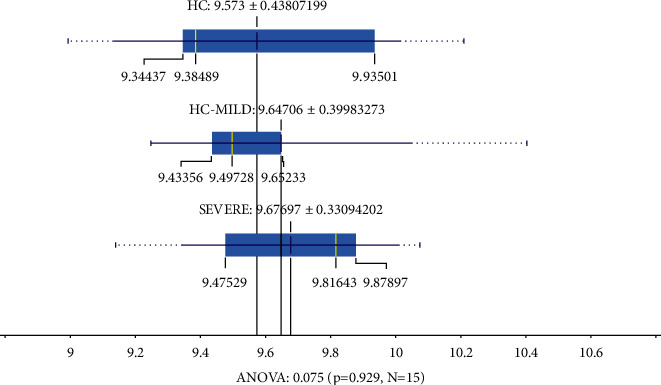
The mean TBK1 expression in the SEVERE and MILD-HC groups not significantly different (*p* ≤ 0.01).

**Figure 13:**
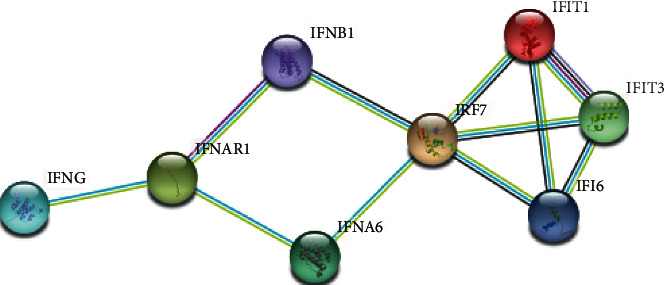
PPI involved in the interferon response obtained from the STRING database with an interaction confidence score of 0.900.

In addition, to understand the IFNG downregulation, we observed the TBK1 (TANK-binding kinase 1-interferon) value, which is an inducer of the IFNG in Figure 12. The TBK expression was not changed in HC, mild, and severe COVID-19 patients, respectively. We also investigated the PPI from interferons-related proteins to understand connections. In Figure 12, we show that all of the genes were connected. From this, we assumed that down or upregulated genes will affect the network. PPI analysis can be seen in Figure 13.

## 4. Discussion

Previous studies reported two molecular functions, two cellular components, and 14 biological processes associated with significant cytokines in the COVID-19 cytokine storm [24]. These GOs were retained from GeneAnalytics (https://geneanalytics.genecards.org/), which can identify gene ontology terms associated with such cytokines. The names of each type of GO can be seen in Table 2.

The clustering results of COVID-19 important protein and GO showed that all GOs of molecular function, all GOs of cellular components, and 9 of 14 GOs of biological processes associated with significant cytokine in the COVID-19 cytokine storm were present in the clustering results. The results of this clustering result had an association with hyperinflammation of cytokine storms in COVID-19. The results of each GO cluster member that are not associated with significant cytokines in the COVID-19 cytokine storm are GOs suspected to be associated with other hyperinflammation in human organs cause of disease complications when exposed to SARS-CoV-2.

Clustering important proteins can develop this research with three types of GOs using a quadripartite graph. Information about the relationship between the three types of GO is needed so that it is hoped that there will be a more in-depth analysis of the relationship between important proteins and three types of GO. As comparison besides GO, we also added enriched data from Wikipathway, which is shown in Table 3.

The severity of COVID-19 was triggered by hyperinflammation of the host. Several factors may drive this, including oxidative stress driven by xanthine oxidase, cytokine productions such as interleukin family, and neutrophil recruitment, triggering microthrombus formations [25]. We followed up by combining gene expression analysis from microarray data collected referred to [21]. The data suggest that IFNAR1 (Interferon-alpha/beta receptor alpha chain), https://www.uniprot.org/uniprot/O14879)IFIT3 (Interferon-induced protein with tetratricopeptide 3, IFIT1 (https://www.uniprot.org/uniprot/O14879)Interferon-induced protein with tetratricopeptide 1, and IFI6 (Interferon alpha inducible protein 6) were upregulated inpatient with severity, while the IRF7 was downregulated (interferon regulatory factor 7).

IFNAR was necessary to activate interferon stimulatory gene (ISG) to suppress the virus to enter the cells. An increase in IFNAR may be associated with the host response to viral infections. Bastard et al. showed that one cause that affected severity was auto antibody IFN type 1 [26].

Moreover, it was found that increasing IFIT1 and IFIT3 has been reported previously in CD16+ monocytes of mild and severe COVID-19 patients, while the IRF7 was not differentially expressed. Interestingly, our analysis showed that the IRF7 was downregulated, while the IFNAR1 was increased. This may reflect to that autoantibody of IFN type 1 may occur and cause IRF7 down expression. On the other hand, besides type 1 IFN, COVID-19 infections were modulated by interferon inflammation that triggered the SARS-CoV-2 entering the cells. It was also reported that interferon response genes were also found to increase as a response to viral infections while the epithelial was infected, including IFI6 similar to our finding. [26–28].

Contribution of interferon type I and type II was previously reported by [29] and related to our results. The analysis of gene expression increased significantly in IFNA6, IFNB1 (*p* < 0.05), and IFNG. An increase of IFNA6 and IFNB1 may reflect activation of hyperinflammation through the type 1 interferon pathway [28]. On the other hand, our data suggested that IFNG was significantly down between mild and healthy control to severe patients. Therefore, downregulation of IFNG may show low antiviral response in the patients and may relate to the severity of COVID-19 patients as shown in Table 4 and Figure 11, respectively (*p* ≤ 0.01). The increase in IFNA6 and IFNB1 is a natural thing because as due to the decrease in IFNG antiviral response, there is no antiviral response that harms the host. Decreased IFNG expression could be decreased IRF7 via TBK1 [29].

## 5. Conclusion

The network analysis, as one of the system biology approach, can help us to reveal the contributing genes to the diseases severity. A combination of enrichment using GOs and transcriptomic analysis showed that hyperinflammation and severity of COVID-19 may be caused by interferon signaling.

## Figures and Tables

**Table 1:** Maximum number of clusters for each type of GO.

Maximum cluster	Number of clusters		
	Molecular function	Cellular component	Biological process
Protein	4	3	7
GO	6	6	27

**Table 2:** GO terms associated with cytokines significant to COVID-19 cytokine storm.

GO type	GO term	Exist in the resulting cluster
Molecular function	1 Cytokine activity	Yes
	2 Growth factor activity	Yes

Cellular component	1 Extracellular space	Yes
	2 Extracellular region	Yes

Biological process	1 Immune response	No
	2 Cytokine-mediated signaling pathway	Yes
	3 Signal transduction	No
	4 Cellular response to lipopolysaccharide	Yes
	5 Inflammatory response	Yes
	6 Positive regulation of cell proliferation	No
	7 Positive regulation of transcription by RNA polymerase II	No
	8 Humoral immune response	Yes
	9 Positive regulation of gene expression	Yes
	10 Positive regulation of tyrosine phosphorylation of STAT protein	Yes
	11 Positive regulation of DNA-binding transcription factor activity	Yes
	12 Cell-cell signaling	No
	13 MAPK cascade	Yes
	14 Negative regulation of the apoptotic process	Yes

**Table 3:** Enriched data from WikiPathway 2021.

Term	*P*-value	Adjusted *P*-value	Genes
Type I interferon induction and signaling during SARS-CoV-2 infection WP4868	2,88*E* − 08	4,00*E* − 06	TBK1; OAS1; IRF3; STAT1; OAS2; STAT2; IRF7; TYK2; JAK1; IFNAR1
Immune response to *tuberculosis* WP4197	9,67*E* − 08	6,72*E* − 05	OAS1; STAT1; STAT2; MX1; TYK2; IFIT1; IFIT3; JAK1; IFNAR1
Host-pathogen interaction of human coronaviruses-interferon induction WP4880	4,54*E* − 06	2,10*E* − 05	TBK1; OAS1; IRF3; STAT1; OAS2; STAT2; TYK2; JAK1; IFNAR1
SARS-CoV-2 innate immunity evasion and cell-specific immune response WP5039	4,29*E* − 02	1,49*E* + 00	IL6; TBK1; IRF3; STAT1; STAT2; MX1; IRF7; JAK1; IFNAR1
Hepatitis B infection WP4666	8,24*E* − 02	2,29*E* + 01	IL6; TBK1; IRF3; STAT1; STAT2; STAT3; IRF7; TYK2; JAK1; IFNAR1
SARS coronavirus and innate immunity WP4912	7,92*E* − 03	1,84*E* + 01	TBK1; IRF3; STAT1; STAT2; TYK2; JAK1; IFNAR1
Non-genomic actions of 1,25 dihydroxyvitamin D3 WP4341	2,04*E* + 01	4,06*E* + 02	IL6; RSAD2; STAT1; OAS2; STAT2; ISG15; TYK2; JAK1
IL-10 anti-inflammatory signaling pathway WP4495	4,59*E* + 02	7,97*E* + 03	IL6; STAT1; STAT2; STAT3; JAK1
Type II interferon signaling (IFNG) WP619	9,95*E* + 02	1,54*E* + 05	OAS1; STAT1; STAT2; IFI6; ISG15; JAK1
Interferon type I signaling pathways WP585	1,09*E* + 05	1,52*E* + 06	STAT1; STAT2; STAT3; TYK2; JAK1; IFNAR1
Type III interferon signaling WP2113	1,52*E* + 06	1,92*E* + 06	STAT1; STAT2; TYK2; JAK1
Overview of interferon-mediated signaling pathway WP4558	2,48*E* + 05	2,88*E* + 06	STAT1; STAT2; TYK2; JAK1; IFNAR1
IL-6 signaling pathway WP364	5,47*E* + 05	5,84*E* + 07	IL6; STAT1; STAT3; TYK2; JAK1
Toll-like receptor signaling pathway WP75	5,88*E* + 05	5,84*E* + 07	IL6; TBK1; IRF3; STAT1; IRF7; IFNAR1
Regulation of toll-like receptor signaling pathway WP1449	3,62*E* + 07	3,35*E* + 07	IL6; TBK1; IRF3; STAT1; IRF7; IFNAR1
Cytosolic DNA-sensing pathway WP4655	9,61*E* + 05	8,35*E* + 07	IL6; TBK1; IRF3; IRF7; ISG15
TLR4 signaling and tolerance WP3851	1,47*E* + 08	1,20*E* + 09	IL6; TBK1; IRF3; IRF7
Interleukin-11 signaling pathway WP2332	9,62*E* + 07	7,43*E* + 08	STAT1; STAT3; TYK2; JAK1
Thymic stromal lymphoPoietin (TSLP) signaling pathway WP2203	1,26*E* + 09	9,23*E* + 08	IL6; STAT1; STAT3; JAK1
IL-4 signaling pathway WP395	2,23*E* + 09	1,55*E* + 10	STAT1; STAT3; TYK2; JAK1

**Table 4:** The expression level of healthy control, mild, and severe COVID-19 patients. Note: Values marked with an asterisk are not significantly different (*p* > 0.05).

ID GENE	HC mean (*N* = 5)	MILD mean (*N* = 5)	SEVERE mean (*N* = 5)	*P*-value	Gene regulation in patient (up/down-regulated)
IFNAR1	6.963 ± 0.313	8.961 ± 0.282^∗^	9.660 ± 0.305^∗^	≤0.01	Up
IFI6	7.739 ± 0.228	10.569 ± 0.739^∗^	10.461 ± 0.505^∗^	≤0.01	Up
IRF7	6.886 ± 0.481	5.796 ± 0.102	4.985 ± 0.376	≤0.01	Down
IRF3	9.699 ± 0.762	8.074 ± 0.288^∗^	7.713 ± 0.274^∗^	≤0.01	Down
IFIT1	3.373 ± 0.247	5.743 ± 0.565^∗^	5.214 ± 0.752^∗^	≤0.01	Up
IFIT3	2.894 ± 0.286	4.387 ± 0.969^∗^	4.699 ± 0.769^∗^	≤0.01	Up
IFNA6	3.140 ± 0.230	5.424 ± 0.272^∗^	5.731 ± 1.114^∗^	≤0.01	Up
IFNB1	2.970 ± 1.208^∗^	3.894 ± 0.461^∗^	6.065 ± 0.244	≤0.01	Up
IFNG	11.731 ± 1.069^∗^	9.729 ± 0.709^∗^	8.273 ± 1.088	≤0.01	Down

## Data Availability

All the data were collected from database such as GO and GEO Browser.
